# Supplementary material for: Modern Carbon–Based Materials for Adsorptive Removal of Organic and Inorganic Pollutants from Water and Wastewater
Source: Molecules. 2021 Nov 1;26(21):6628. doi: 10.3390/molecules26216628 (PMC8587771; doi:10.3390/molecules26216628)
Supplement: Supplementary file 1 [file molecules-26-06628-s001.zip › molecules-1301684-supplementary.pdf]

Supplementary Material for the Review

Modern Carbon–Based Materials for Adsorptive Removal of Organic and Inorganic Pollutants from Water and Wastewater

Vera I. Isaeva [1,*], Marina D. Vedenyapina [1,*], Alexandra Yu. Kurmysheva [1], Dirk Weichgrebe [3], Rahul Ramesh Nair [3], Ngoc Phuong Thanh Nguyen [3], and Leonid M. Kustov [1,2,*]

[1] N. D. Zelinsky Institute of Organic Chemistry, Russian Academy of Sciences, Leninsky prospect 47, Moscow 119991, Russia;
[2] Chemistry Department, Moscow State University, Leninskie Gory 1, bldg. 3, Moscow 119992, Russia;
[3] Institute for Sanitary Engineering and Waste Management, Leibniz University Hannover, Welfengarten 1, D-30167 Hannover, Germany
[*] Correspondence: veraisaeva2019@mail.ru (V.I.I.); mvedenyapina@yandex.ru (M.D.V.); lmk@ioc.ac.ru (L.M.K.)

List of tables **Pg no.**

S1 Summary of MAB and pristine biochar from collected literature 2

S2 Adsorption mechanisms of heavy metals and organic compounds on biochar. 7

S3 Some salient studies on application of biochar for adsorption 9

Table S1: Summary of MAB and pristine biochar from collected literature

**Biochar Feedstock**	**HTT (℃)**	**BET (m^2/g)**	**pH**	**Proximate Analysis (%Dry Wt.)**				**Ultimate Analysis (% Dry Wt.)**					**Ref**
				**Ash**	**VM**	**Moisture**	**FC**	**C**	**H**	**N**	**S**	**O**	
Sewage sludge	550		8.4	60.95				28.77	4.62	3.17	0.42		[425]
**Digestate from the anerobic digestion of wastewater treated with Al to precipitate P from wastewater**	250		5.56	29.59	48.41	2.92	16.16	38.2		0.175			[424]
	350		5.39	40.92	29.51	2.97	23.63	37.35		0.188			
	450		7	46.98	20.9	2.68	26.75	36.77		0.185			
	550		7.95	51.08	13.84	2.77	29.55	35.85		0.166			
Poultry litter	300	7.73	8.4	58.7	23.2	1	17.1	32.3	2.91	2.93			[442]
	400	29.94	9.7	62.1	13.4	1.2	23.3	26.6	2.18	2.4			
	500	33.64	10.3	66.5	7.2	1.2	25.1	19.1	0.98	1.62			
Spent Agaricus bisporus (Mushroom)	350	36.2	8.83	66.5				17.53	1.32	1.48		13.49	[422]
	450	48.74	9.37	71.76				15.91	0.82	1.2		11.73	
	550	64.19	9.51	74.08				15.22	0.54	1.06		9.87	
	650	101.39	11.38	77.23				14.53	0.37	0.88		7.87	
	750	37.08	11.82	82.12				12.17	0.26	0.85		4.65	
**Green waste from tomato crop**	550		12.1	56.2				31.2		2.6	4.1		[407]
Rice husk	600	10.995	7.8	41.96	35.67		17.34	37.91	2.06	0.89	2.06	59.14	[443]
Paper sludge/wheat husk	500	63.8	8.3	40.2				50.5	1.55	1.29	0.14	6.4	[444]
Digestate from biorefinery plant	500			49.55	20.18		30.27	35.93	1.75	2.7	0.47	9.29	[445]

Municipal solid wastes	400	20.7	8	6.1	22.3			65.2	48.6	12.2	1.3	0.1	31.7	[436]
	500	29.1	8.5	9.2	26.4			63.8	59.5	9.1	1.4	0	20.8	
	600	29.8	9	6.2	15.1			78.2	70.1	8.4	1.3	0.1	13.7	
Food waste solid digestate	400	4.73		34.27	36.24			29.49	46.89	2.12	1.9	0.16	14.66	[446]
	500			36.31	27.16			36.53	48.34	1.53	1.85	0.12	11.85	
	600			36.94	16.45			46.61	50.41	1.26	1.86	0.09	9.44	
	700			37.66	14.09			48.25	50.21	1.13	1.82	0.08	9.1	
	800	462.82		37.92	13.83			48.25	50.19	1.09	1.76	0.09	8.92	
Diary manure digestate	350	1.43	8.24	67.33	32.67				49.82	0.66	0.16		49.36	[447]
	450	7.36	9.49	73.76	26.24				35.11	2.04	0.04		62.82	
	550	3.23	10.4	79.27	20.73				19.87	0.49	1.63		78.02	
Municipal WWTP Sewage sludge 1	200			67.5					17	3.21	2.48	0.21	9.58	[448]
	300			75.7					14.7	1.77	2.33	0.33	5.22	
	500			86.6					8.9	0.77	1.1	0.33	2.3	
	700			89.9					7.98	0.22	0.54	0.49	0.88	
Municipal WWTP Sewage sludge 2	200			61.7					16.9	3.58	2.53	0.46	14.9	[448]
	300			69.7					16.2	2.38	2.53	0.36	8.78	
	500			85.4					8.43	0.91	1.07	0.43	3.78	
	700			89.3					7.43	0.71	0.64	0.45	1.52	
Pig manure	600	15.56		46.52	19.11		5.18		44.13	2.49	2.09			[449]
Swine solids	350	0.92	8.4	32.5	49.8	-		17.7	51.51	4.91	3.54	0.8	11.1	[450]
	700	4.11	9.5	52.9	13.4			33.8	44.06	0.74	2.61	0.85	4.03	
Turkey litter	350	2.6	8	34.8	42.1			23.1	49.28	3.6	4.07	0.55	15.4	[450]
	700	66.7	9.9	49.9	20.8			29.2	44.77	0.91	1.94	0.41	5.8	

Swine Manure	400	4.91	10.95	49.8	35.47	1.98		7.49		0.44			[451]
Chicken manure	350			52	36.9		11.1	31.2	1.97		0.31	10.9	[452]
	450			55.3	30.6		14.1	27.2	1.92		0.44	11.4	
	750			56.4	26.5		17	24.7	0.67		0.29	16.3	
Waste mushroom	400	3.36	8.95	32.33	-	-	-	34.46	-	2.36	-	-	[453]
	500	2.72	9.24	41.08	-	-	-	38.46	-	1.76	-	-	
	600	1.97	9.4	47.08	-	-	-	39.96	-	1.28	-	-	
	700	1.71	10.28	54.34	-	-	-	41.08	-	1.17	-	-	
Digested Sewage sludge	300	-	5.32	52.8	33.8	4.3	9.1	25.6	2.55	3.27	-	8.33	[454]
	400	-	4.87	63.3	25.7	4.2	6.8	20.2	1.28	2.4	-	4.61	
	500	-	7.27	68.2	20.7	3.5	7.6	20.3	0.88	2.13	-	0.65	
	700	-	12	72.5	15.8	3.4	8.3	20.4	0.51	1.2	-	0	
Sewage sludge	350	-	4.49	51.24	-	-	-	24.35	1.73	3.17	-	75.23	[455]
	500	-	5.21	65.81	-	-	-	20.99	0.88	2.9	-	70.75	
Tomato green waste	550		11.21	56.2	-	-	-	32.3	1.24	2.04	-	-	[456]
Blue mallee (Eucalyptus polybractea)	550		9.63	22.3	-	-	-	54.9	2.16	1.39	-	-	[456]
Industrial sludge	600		8.4	92.3	1.2		6.5	9.5	0.4	1.5	2.2		[457]
Waste rubber wood sawdust	450			14.5				82.3	3.2	0.4		14	[458]
	550			16.3				90.6	2.5	0.3		6.6	
	650			17.8				92	1.7	0.2		6.2	
	750			19.5				97.3	1.1	0		1.2	
	850			20				93.4	1.3	0.1		0.5	
Mallee wood	300			0.7	59.5	4.6	35.3	60.3	5.3	0.18	0.02	34.14	[459]
	320			0.9	39.3	2.9	56.9	72.9	4.6	0.24	0.02	22.21	
	330			1.1	37	2.7	59.2	73.6	4.5	0.27	0.03	21.52	

	400			1.2	24.4	4.5	69.9	79.1	3.7	0.29	0.04	16.8	
	450			1.4	19.3	4	75.3	82.9	3.3	0.32	0.03	13.38	
	500			1.3	14.4	4.5	79.8	85.5	3	0.34	0.03	11.14	
Pinecone	500	192.97	6.77	8.96				74.64	2.62	1.81		20.94	[460]
Walnut shell	900	227.1	9.7	40.4				55.3		0.47	9.4		[461]
Straw and stalk of rapeseed plant	400	16.036		12.22	27.06		60.72	71.34	3.93	1.43	0.24	10.84	[462]
	500	15.695		12.91	17.49		69.6	75.03	2.62	1.41	0.24	7.79	
	600	17.579		13.85	11.49		74.66	78.48	1.88	1.53	0.32	3.94	
	700	19.258		14.37	8.96		76.67	79.48	1.2	1.35	0.31	3.29	
	800	19.014		15.32	6.05		79.68	79.51	0.72	1.45	0.39	2.61	
	900	140.41		16.12	3.55		80.33	79.86	0.42	1.57	0.36	1.67	
Orange peel	250	51.6		3.17				56.5	5.11	1.69			[463]
	400	281		6.93				65.7	3.46	1.8			
	700	501		14.9				67	1.47	2.05			
Bamboo (D. giganteus Munro)	500			3.9	8.1	6.5	81.5	82.1	2.72	0.54	0.001	14.6	[464]
Sugarcane leaves/trash	450	8.1	8.26	36.9				42	2.51	0.9	0.154	46.4	[465]
	550	58.9	8.6	35				44	2.31	1.08	0.154	15.5	
	650	178.5	9.18	41.5				51	1.83	0.71	0.136	11.3	
	750	171.3	9.63	47.8				55.5	1.27	0.71	0.131	0.9	
Sugarcane bagasse	450		7.15	35.3				37.5	1.8	0.58	0.078	60	[465]
	550	151.3	8.54	36.3				39.5	1.34	0.57	0.084	23.2	
	650		8.66	37.6				54.1	1.5	0.44	0.036	7.7	
	750		8.81	39.6				56.3	1.12	0.38	0.016	4.6	
Corn Stover	300	3.19	7.7	5.7	54			45.5	5.4	0.63		42	[466]
	400	3.17	8.8	12.5	45.5			64	3.9	0.42		32	
	500	4.58	9.7	18.7	33.8			64.5	2.7	0.25		33	

Switch grass	Btwn 500-700	67.1	9.33	23.8	6.6		69.5	71.7	1.29	0.9		2.1	[467]
Public Miscanthanus	500	0.25	9.57	8.72	16.69		74.59	77.72	2.94	1.08	0.38	17.89	[468]
	700	239.24	10.61	9.94	11.44		78.62	79.3	2.64	1.45	0.1	16.51	
	900	783.74	8.96	10.85	9.28		79.87	79.24	2.01	1.55	0.08	17.12	
Pine bark	550		8.3	2				83	3.41	0.3			[469]
Peanut shell	565	1224		5.85	24.49	6.81	62.85	43.66			0.31	24.88	[470]
Chonta pulp	630	652.8		10.48	34.62	5.98	48.92	63.11			3.13	22.66	[470]
Corn cob	600	778.3		4.22	19.99	5.23	70.22	54.4			0.02	27.82	[470]
	350			12.9	34.6		52.5	60.5	3.92		0.09	19.5	
Coffee husk	450			12.9	26.2		60.9	61.3	3.65		0.1	19	[452]
	750			19.6	17.6		62.8	66	1.57		0.23	9.8	
Rapeseed	300	6.96	9.16	12.34	35.8	-	-	47.35	4.49	1.59	-	24.4	[471]
	500	18.35	9.98	18.7	23.27	-	-	55.46	2.86	1.2	-	20.2	
Rice straw	300	8.13	9.4	15.41	32.95	-	-	49.28	4.26	1.43	-	27.2	[471]
	500	32.51	10.85	24.21	18.73			58.77	3	1.1	-	21.9	
Eucalyptus bark	350		6.18	3.95				55.81	3.83	0.46		39.9	[455]
	500		5.65	3.25				74.06	2.41	0.66		22.87	
Banana peel	500	51		10.5	15.5		67.6	58	6	1.3	0	34.7	[472]
Cypress saw dust	500	205	7.34	1.5	27	3.6	68	84.7	3.5	0.3	0.05	11.6	[409]
Wood	600	273.62	9.8	5.87	8.92		80.2	86.3	0.79	0.73	0.22	12.18	[443]
Mixed wood	600	335	8.41	6.6				78.12	2.44	0.81	-	11.5	[473]
Bagasse	450	15.3	8.86	7.35				74.4	2.51	-	-	19.7	[474]
Hickory chips	400	12.9	9.92	2.32				83.7	1.11	-	-	13.8	[474]
Coconut shell	500	212	8.8	5.53				86.6	3.03	0.08	-	10.3	[475]
Rice straw	800	70.4	10.37	31.75				57.01	3.23	1.48	-	13.82	[476]
Sewage sludge	800	27.29	7.75	89.78				7.63	0.53	1.18	-	6.88	[476]

Sewage sludge	700	25.68	-	46.55		80.17		15.55	0.86	1.18	0.51	35.35	[477]
Sewage sludge + bamboo waste	700	47.48	-	-		6.43		22.7	0.82	1.07	0.62	36.91	[477]
Sewage sludge	850		-	33.35	60.53	78.55		36.88	5.02	5.03	1.17	52.01	[478]
Hazelnut shell	850		-	1.23	76.57	8.22		48.11	2.62	0.52	3.07	45.73	[478]
Paddy straw	500	45.8	10.5	52.37	6.46	2.07	39.1	86.28	3.12	3.25		7.35	
Cocopeat	500	13.7	10.3	15.9	14.3	2.55	67.25	84.44	2.88	1.02		11.67	

Table S2: Adsorption mechanisms of heavy metals and organic compounds on biochar

Mechanism	Heavy metals	Ref.
**Surface sorption**	Referred as physisorption, a diffusional movement of metal ions into biochar surface pores without forming chemical bonds. These attraction weak forces are called *van der Waals* forces. The value of surface areas and pore volumes are proportional to the amount of adsorbed metal ions on the biochar surface, resulting from the increasing pyrolysis temperature	[479]
**Cation exchange**	The cation (i.e. ion) exchange takes place on the biochar surface when the dissolved metal ions that have a positive charge are being altered selectively by other target metal ions. This replacement is based on the same characteristics of ionic radius, charge differences and bond characteristics of most metals from group 1 to 3 of the periodic table.	[480]
	The capacity of cation exchange (CEC) is modified by surface functional groups, with a higher value of CEC resulting in better heavy metal removal. High O contents and acidic surface sites have also been reported to have higher CEC.	[481]
**Precipitation**	Immobilization of heavy metals is also referred to as precipitation mechanism. The intermediate ionization potentials of elements range from 2.5 to 9.5 eV, the metals are prone to precipitate on the surface of biochar.	[480]

	Through pyrolysis, biochar is mostly formed with alkaline feature that may activate the metallic precipitation mechanism. A case study showed that biochar with pH of 10.93 had isolated Pb via hydrocerrusite[1] formation.	[482, 483]
**Electrostatic interactions**	A surface charged biochar interacting with metals ions is considered as electrostatic attraction. The pH value of solution and biochar's point of zero charge (pH_{PZC} or pH-Zeta potential) will determine the widespread presence of this interaction.	[484, 485]
**Complexation**	Complexation of metallic species is the binding mechanism that comprises multi-atom configurations with certain metal-ligand interactions, especially for the transition metals that have d-orbitals partially filled and high affinity for a ligand.	[486]
	In low-temperature pyrolysis of biochar production, heavy metals have been reported to be efficiently bound by oxygen functional groups.	[487, 488]
	A research paper has concluded that metallic complexation from plant-derived biochar has a higher probability of complex formation than biochar produced from animal-related products.	[479]

**Mechanism**	**Organic compounds**	**Ref.**
**Electrostatic interactions**	The electrostatic attraction will happen when a negatively charged biochar surface meets positively charged organic compounds. The sizes and spacing of atomic particles will determine the strength of this interaction.	[489]
**π- π electron-donor acceptor interactions**	Biochar surface groups (e.g. carboxylic acid, nitro, and ketonic) perform as electron acceptors and form *π*- *π* interaction with aromatic molecules, enhancing the adsorption of these particles.	[490]
**Complexation**	The surface complexation mechanism follows *van der Waals*model, in which the charged surfaces interact mutually in a self-created electric field.	[491, 492]
	One of the case studies showed that methyl orange was removed by ferric oxide-biochar with complexation in single solute solution environment.	[493]

[1] A secondary mineral usually found in oxidized lead ore deposits

	Other research reported the elimination of doxycycline by copper impregnated biochar, due to the reaction between copper surface complexes and oxygen functional groups that are activated on biochar surface.	[415]
**Pore disffusion**	This pore-filling mechanism happens when organic pollutants were captured on biochar surface (with mesopores of 2-50nm ad micropores < 2nm). The process is affected by pore size distribution of biochar, organic molar mass and polarity. Biochar that consists small amount of volatile substances can archive high adsorption efficiency from pore-filling, at low organic concentration.	[494]
**Partitioning**	The process occurs when organic adsorbate diffuses into non-carbonized areas of biochar surface. The characteristics of non-carbonized portion (e.g. crystalline or amorphous sources) and carbonized fractions of biochar will determine the adsorption effect.	[494]
	Biochar that has high amount of volatile substances can archive high adsorption efficiency from partitioning mechanism, at high organic concentration.	[495]
**Hydrophobic interactions**	Biochar that has low surface oxidation will likely react with hydrophobic organic compounds. This is one of the ways to eliminate organic contaminants due to biochar hydrophobicity.	[438]
**Hydrogen bonding**	Surface functional groups containing oxygen can interact with N-containing and F-containing functional groups to form H bonds.	[496]

Table S3: Some salient studies on application of biochar for adsorption.

Adsorbents		Adsorbates		Adsorption		Note	Reference
Biomass source	Temp. (°C)	Types	Source	Capacity (mg/g)	Mechanism		

**Wood/ Plant based**	Mixed wood	600	COD	WW	-	Electrostatic, π- π, hydrophobic interactions	Treatment efficiency: 75.5%	[473]
	Pine wood	700	BOD_5<br>Cr^{2+}<br>Pb^{2+}	LL	-	Cation exchange, pore diffusion	Optimal dose for treatment of:<br>BOD_5: 6.05g/100ml,<br>Cr, Pb: >13.25g/100ml	[497]
	Gliricidia sepium wood	700<br>500<br>300	Crystal violet	AS	125.53<br>23.71<br>11.02	Pore diffusion, π- π interaction, hydrogen bonding, electrostatic interactions	Adsorption capacity maximum at pyrolysis temperature-700, pH-3	[498]
	Hickory chips	400	Hg^{2+}	AS	5	Hg- π coordination, complexation, ion exchange	Adsorption capacity recorded at the highest Hg^{2+} initial concentration (5mg/L)	[394]
**Agricul-ture**	Bagasse	450	Hg^{2+}	AS	13	Hg- π coordination, complexation, ion exchange	Adsorption capacity recorded at the highest Hg^{2+} initial concentration (5mg/L)	[394]
	Coconut shell	500	Cd^{2+}<br>Pb^{2+}	AS	3.5<br>13.4	Cation exchange	Unit of adsorption capacity is converted from mmol/kg to mg/g	[475]
	Pecan nutshell	800	Reactive red 141	AS	130	Chemical adsorption	Treatment efficiency: raw material-23%, biochar-85% with pH of 3	[499]
	Wheat straw	500	As^{3+}<br>P<br>Cr^{6+}	AS	16.21<br>125.4<br>12.23	Electrostatic interactions, physical sorption	Impregnated adsorbent by Bismuth	[500]
	Exhausted olive pomace	300	Ni^{2+}	AS	$27.6*10^{-6}$	Ion exchange, complexation, π- π interaction, precipitation	Treatment efficiency: 85.5%	[501]

	Rice straw	800	Cd^{2+}	AS	58.65(*)<br>42.48(**)	Precipitation, complexation, C- π coordination, cation exchange, physical sorption	(*) non-magnetic adsorbent<br>(**) magnetic adsorbent by chemical co-precipitation	[476]
**Urban organic wastes**	Sewage sludge	800	Cd^{2+}	AS	7.22(*)<br>4.64(**)	Precipitation, complexation, C- π coordination, cation exchange, physical sorption	(*) non-magnetic adsorbent<br>(**) magnetic adsorbent by chemical co-precipitation	[476]
	Sewage sludge	550	Cu^{2+}<br>Cd^{2+}	AS	89.98<br>114.68	Ion exchange, complexation, electrostatic attraction, metal- π interaction	Modified adsorbent by hydroxyapatite (HAP)	[502]
	Sewage sludge	300	Ni^{2+}	AS	$37.8*10^{-6}$	Ion exchange, complexation, π- π interaction, precipitation	Treatment efficiency: 96.8%	[501]
	MSW-Organic fraction	300	Ni^{2+}	AS	$35.7*10^{-6}$	Ion exchange, complexation, π- π interaction, precipitation	Treatment efficiency: 94.7%	[501]
	Sewage sludge	400	Cd^{2+}<br>Pb^{2+}	AS	861.11<br>2931.76	Ion exchange, complexation, electrostatic attraction, co-precipitation	Synthesized by Mg(II)	[503]
**Mix wastes**	Paper sludge & wheat husk	500-600	2,4-dichlorophenol	AS	9.28	Electrostatic, π- π interactions		[444]
	Sewage sludge & bamboo waste	700	Ciprofloxacin	AS	62.48	π- π interactions, H-bonding, ion exchange, complexation		[477]
	Sewage sludge & hazelnut shell	850	Cu^{2+}	AS	42.28	Complexation		[478]
